# Influence of Osteoporosis on the Course of Apical Periodontitis

**DOI:** 10.1055/s-0044-1785533

**Published:** 2024-05-17

**Authors:** Evgeni Stanev, Radosveta Ivanova Vasileva

**Affiliations:** 1 Department of Conservative Dentistry, Faculty of Dental Medicine, Medical University of Sofia, Sofia, Bulgaria

**Keywords:** osteoporosis, apical periodontitis, corticosteroids, bisphosphonates, bone resorption, calcitonin, raloxifene, alendronate, zoledronate

## Abstract

Osteoporosis is a disease characterized by disruption of the bone microarchitecture. It is observed in both sexes, but to a greater extent in women. It affects the whole body, including the jaws. The main indicator of the presence of osteoporosis accepted by the World Health Organization is bone mineral density. The aim of this article is to find data on the influence of osteoporosis on apical periodontitis, to investigate how the intake of osteoporosis drugs affects apical periodontitis, and to establish various data that may be of benefit to the dental practitioner when treating patients with osteoporosis and apical periodontitis. Open-access publications are included. The presence of osteoporosis is important to the dentist. Apical periodontitis in these patients has a faster progression. They are characterized by inflammation and destruction of the tissues located around the tooth root. Osteoporosis has a destructive effect on bone tissue through different mechanisms: nuclear factor-κβ ligand and NLRP3/Caspase-1/IL-1β cascade. It is also associated with low estrogen levels. Various medications such as corticosteroids, bisphosphonates (alendronate, zoledronate (Zoledronic acid), calcitonin, raloxifene, and strontium used to treat osteoporosis can affect the course of apical periodontitis. When treating patients with periapical lesions, the dentist must take a proper medical history and general medical history. In cases of osteoporosis or taking bisphosphonates and other medications, consideration should be given to whether consultation with a specialist is necessary, what treatment approach would be most appropriate, and what the prognosis will be. Chronic diseases affect both the general state of the body and dental health. It has been found that in patients with osteoporosis, inflammation of the apical periodontium develops with faster bone resorption. Before starting dental treatment, it is important to specify the etiology of osteoporosis, the bone density of each patient, as well as the medications they are taking.

## Osteoporosis

Osteoporosis is a disease characterized by reduced bone mass and disruption of bone microarchitecture. Bone strength is compromised and the risk of fractures is increased. Osteoporosis is the most common bone disease in humans and represents a significant public health problem. Although it affects a large number of patients of both sexes and all races, it is more common among Caucasians, women, and the elderly. It is a silent disease until fractures occur. 1

When examining the bone density of patients aged 50 and over, the incidence of osteoporosis increases with age: from 3 to 4% to 30% in 80-year-old patients. 2 3 In a 2021 study, about 18% of the world's population is reported to be affected by this disease (23% women and 12% men). 4

The main indicator of the presence of osteoporosis accepted by the World Health Organization is BMD (bone mineral density). It is measured using X-rays by determining the amount of mineral structures absorbing the X-rays. Values equal to or above −1 are considered normal. With values from −1 to −2.5, the patient has reduced bone density, and below −2.5 a diagnosis of osteoporosis is made. 5

**Fig. 1:**
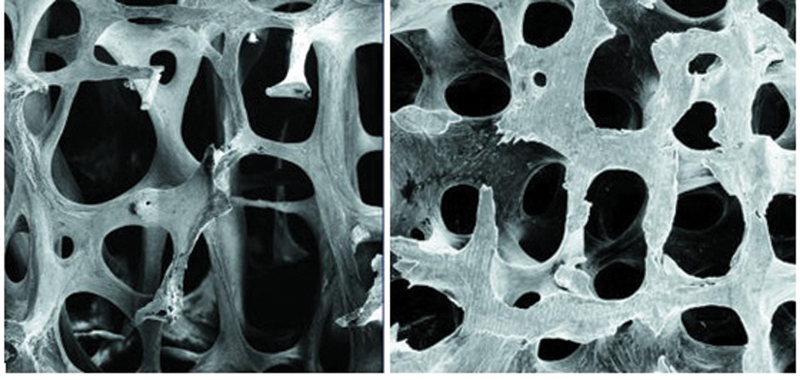
Difference in cancellous bone: in the first photo (left)—Thinned bone trabeculae with defects and wide intertrabecular spaces are seen in patients with osteoporosis. In the second photo (on the right)—normal cancellous bone (courtesy of the BMJ Publishing Group Ltd). 6

Thinned bone trabeculae with defects and wide intertrabecular spaces are seen in patients with osteoporosis ( Fig. 1 ). Osteoporosis is classified as primary and secondary. Primary osteoporosis is type 1 and type 2. Type 1 is known as postmenstrual osteoporosis, caused by estrogen deficiency, mainly affecting trabecular bone. This is the reason why women are more often affected than men. Type 2 is senile osteoporosis. In it, the loss of bone mass is due to the aging of the cortical and trabecular bone structures. Secondary osteoporosis is the result of diseases, medication or sudden lifestyle changes. 6 7 8

Osteoporosis demonstrates several stages: 1. Osteopenia (decreased bone density); 2. Incipient osteoporosis with risk of fractures; 3. Pronounced osteoporosis with occurring fractures (most often at the femoral neck, vertebrae, and wrists).

A family history of such a fracture is considered a risk factor for osteoporosis. Fractures of the hip, wrists, and spine can also be included here. 9 Fractures can also be due to other risk factors, even with normal levels of bone density, most notably the age of the patient. 10

The dentist may detect reduced bone density during an X-ray examination and this may be a reason to refer the patient for a consultation regarding suspected osteoporosis. This is usually done during orthopantomographic imaging. In the reading, attention is directed to the intensity of the shadow of the mandibular cortex relative to that of the trabecular bone. The area below the foramen mentalae is most suitable, due to the lack of muscles or muscle connections, and the fact that the distance between the foramen and the lower edge of the cortex remains constant, regardless of life expectancy and resorption of alveolar bone. 11

The loss of BMD in various sites of the body, including the mandible, is the main sign of osteoporosis. Thus, the computer-aided diagnosis system is developed for bone density assessment and patients were classified into normal, osteopenia, and osteoporosis groups using a digital panoramic radiograph. A computer-aided system can be used for osteoporosis and osteopenia assessment by measuring the mandibular cortical width and texture on dental panoramic radiographs with the average system accuracy of 89.52%. 12

Osteoporosis can be associated with diseases of the oral cavity. Most often, the dentist encounters patients who report the presence of primary osteoporosis, especially when treating postmenopausal women.

## Apical Periodontitis

Apical periodontitis is a common inflammatory disease of the periodontium that affects a wide range of population all over the world. It is known that periodontitis is a host-mediated process, which includes both periodontal tissue destruction and bone resorption.

In periodontitis, the pathogen releases chemicals that activate the white cells of the innate immune system to produce proinﬂammatory cytokines that play a vital role in the progression process of periodontitis. Moreover, these pathogens can activate the acquired immune system that in turn contributes to more and more progression of the inflammatory condition. As the immune response continues, released cytokines and chemokines can cause severe damage to the gingiva, periodontal ligaments, and alveolar bone. This causes permanent alveolar bone damage. Cytokines play a vital role in the inflammatory process of periodontitis such as interleukin-1β (IL-1β), IL-6, tumor necrosis factor-α (TNF-α), and receptor activator of nuclear factor kappa-Β ligand (RANKL). 13

## Apical Periodontitis and Osteoporosis Pathology

### Low Estrogen Levels

Osteoblasts, osteoclasts, and osteocytes have estrogen receptors (ERs). When binding to them, the production of proteins is regulated. On the other hand, estrogen and steroid hormones affect the body's immune response.

Brasil et al investigated the influence of estrogen on the development of periodontitis in experimental mice over a long period. The mice were initially ovariectomized and allowed 120 days to reach low estrogen levels. This is followed by pulp exposure of first molars to induce periodontitis. The results showed significantly larger periapical lesions compared to the control group. Serum calcium concentration was similar in both groups. Alkaline phosphatase was higher compared to the control group, but without statistically significant differences. 14

### Receptor Activator of Nuclear Factor Kappa-Β Ligand

Estrogen decreases receptor activator of nuclear factor-κβ ligand (RANKL) levels and increases osteoprotegerin, thereby decreasing osteoclast activity. Furthermore, estrogen reduces osteoclast differentiation and facilitates osteoclast apoptosis via transforming growth factor-beta. The hormone also affects osteocytes. Without it, they cannot respond normally to mechanical pressure from periodontal inflammation. Osteocytes can also synthesize RANKL. At low estrogen levels, IL-7, T-cells, and B-cells of the immune system are also activated, which help bone resorption. 15

Zhang et al investigated how osteoprotegerin levels affect periapical lesions. Mice with low estrogen levels and artificially induced periodontitis were used. The authors found that more significant bone involvement was observed with increased RANKL expression as a consequence of estrogen deficiency. In parallel, an increase in osteoprotegerin was also observed, as a counter-protective reaction. 16 17 18

Similar were the results of Ikeda et al: Anti-RANKL antibody reduced the number of osteoclasts in periapical tissues. 19 A lack of estrogen receptor alpha (ERα)-mediated suppression of RANKL expression was observed in increased bone resorption. 20

RANKL levels also depend on the patient's psychological state. This should also be taken into account in patients with osteoporosis. 21

### NLRP3/Caspase-1/IL-1β Cascade

Levels of substances involved in the NLRP3/Caspase-1/IL-1β cascade are increased in affected tissues of patients with reduced estrogen levels, in contrast to patients with normal levels. Similar data have been obtained in animal studies. Nod-like receptors (NLR) play an important role in autoimmune and inflammatory diseases. There are 22 types, 14 of which are NLRP (nucleotide-binding domain and leucine-rich rep protein). Of these, 5 subtypes (NLRP1, NLRP3, NLRP6, NLRP7, and NLRC4) are involved in the formation of IL-1β, with NLRP3 being the most important. 22 It can be activated by various stimuli, such as bacterial peptidoglycans, lipopolysaccharides, endogenous proteins of damaged cells, and others.

Upon activation, it forms an oligoprotein with apoptosis-associated proteins and procaspase-1, which in turn activates the caspase-1 cascade. As a result, the inflammatory cytokines (interleukins), IL-18 and IL-1β, are formed.

Various factors can affect IL-1β levels: genetics, systemic diseases, intake of herbal extracts. IL-1β +3954 gene polymorphism is associated with periodontitis and is expected to be among the several causes of respective pathology. 23

The severe acute respiratory syndrome coronavirus 2 (SARS-CoV-2) infection can trigger a profound cytokine response in the host, often referred to as a “cytokine storm” that is exhibited as elevated serum levels of TNF-α and IL-1β with higher levels in those who are critically ill. 24 Centella asiatica is one of the plants that is widely known as medicinal herb. The extract of it has the effect of reducing the proinflammatory cytokine IL-1β via inhibiting NF-κB. 25

TNF and IL-1β levels are the two main indicators by which inflammation can be determined in apical periodontitis. 26 The levels of IL-1β in the unstimulated saliva are also higher in patients with periodontitis compared with the healthy control and gingivitis group. 27 This indicates that this IL-1β is an important element of the inflammatory process in apical periodontitis.

Cascade of reactions leads to bone resorption as a consequence of the inflammation caused by apical periodontitis. 28 29 When studying estrogen-deficient rats, it was found that this cascade of reactions was activated to a greater extent. 30

In support of this theory is a study on 50 experimental mice, the ovaries of half of them were removed, and the pulp of their first molar was exposed. As early as the first week, serum estrogen levels drop significantly, and osteoclast levels in the bones increase. NLRP3/Caspase-1/IL-1β levels were also increased in the low estrogen mice. The authors believe that pharmacological suppression of NLR signaling may be beneficial in the treatment of inflammatory diseases in menopausal women. 31

### Matrix Metalloproteinases and Cathepsin K

Romualdo et al investigated the levels of proinflammatory cytokines MMPs (matrix metalloproteinases) and cathepsin K in periodontitis of ovariectomized mice. The results showed increased levels of IL-1β, TNF-α IL-6, MMP-8 and MMP-13 compared to the control group. 32 Bahuguna et al also noted a role for cathepsin K in osteoclastic bone resorption. It is inhibited by odanacatib, a drug used to treat osteoporosis. In patients on such therapy, endodontic treatment can be performed with normal bone regeneration around the apex. 27 33

Certain molecules like cathepsin, found at a high level in periodontal tissues, particularly in patients with periodontitis, are involved in the mechanism of entry of SARS-CoV-2 into cells. Periodontopathic bacteria could also play a direct role in the mechanism of entry of SARS-CoV-2 by cleaving the S-protein, and the cytokines produced during periodontitis could add to the cytokine storm found in the severe forms of coronavirus disease 2019. 34 In this way, ARS-CoV-2 can affect patients with osteoporosis and apical periodontitis.

## Age and Postmenopausal Period

Guan et al investigated the mechanism of estrogen action on periapical changes. They observed the periapical tissues after extraction of teeth with periodontitis in 30 women, half under 35 years of age, and the other half over 58 years of age. Women with immune diseases, diabetes, and other endocrine diseases were not included. After extraction, the tissues were processed and subjected to immuno-histo-chemical analysis. The levels of NLRP3, caspase-1, and IL-1β of postmenopausal women were significantly higher than those of other women. The levels of the precursor proCaspase-1 were also increased, and the authors believe that its conversion to caspase-1 is regulated by estrogen. 31

Lucisano et al note that in patients with reduced estrogen levels, the microbiota also changes, leading to a more significant progression of periapical lesions. 35 Therefore, in patients with osteoporosis as a result of the onset of menopause and the need for the treatment of periapical lesions, the change in the composition and quantity of microorganisms in the lesions should be taken into account.

Rossetti et al analyzed the publications related to periapical changes in postmenstrual osteoporosis and how it affects the development of periapical lesions. All 12 studies that the authors analyzed confirm the fact that low estrogen levels favor the development of periodontitis. More than half of them is based on histomorphological analysis of bone loss in the studied area. In five of them, the radiographic image was analyzed. Microcomputed tomography was used in three patients, fluorescence microscopy in two, and radiographic density assessment in one patient. Every single study emphasizes that at low levels of estrogen, bone resorption increases compared to the control group. The explanation is related to various proinflammatory cytokines, such as IL-1, IL-6 and TNF-α. 36 37

In a study by López-López et al, it was found that 25% of postmenopausal women with osteoporosis had at least one periapical lesion, compared to 7.4% in the nonosteoporotic group. A statistically significant relationship was found between bone density and the frequency of periapical lesions. 38

In a study by Duncea et al, 97 postmenopausal women were included, dividing them into two groups: with and without osteoporosis, focusing on bone density of L1-L4 vertebrae, femur, and mandible. The results demonstrate that in patients with osteoporosis, the risk of periapical lesions is six times greater. Its size increases as the patient's bone density decreases. 39

## Medication

Taking various medications in connection with osteoporosis can have an impact on the development of periapical processes.

### Corticosteroids

Corticosteroids can suppress osteoblast functions. However, there is still uncertainty about the exact mechanism of action. They affect calcium absorption, inhibit osteoblast protein production, osteoblast apoptosis, and prolong the life of osteocytes. They also affect the expression of RANK and osteoprotegerin and IL-6, as well as other cytokines leading to bone resorption. Glucocorticosteroid-induced osteoporosis is thought to occur in 0.5 to 1% of the general population, depending on the doses that are used. The most significant bone loss was observed after the sixth month of admission. 15

### Bisphosphonates

Means of choice for prevention and treatment are bisphosphonates—alendronate, zolendronate (ZOA), risedronate, and ibandronate. A study by Hsiao et al confirmed that bisphosphonates can affect healing of the periapical lesion, both in primary endodontic treatment and in retreatment. 40 Sampaio et al consider that their action supports the healing process during endodontic treatment. 41

Bisphosphonate intake was associated with significantly fewer periapical lesions among patients with osteoporosis. Bisphosphonates suppress bone resorption by interfering with the internal cellular enzyme system of osteoclasts. 42 In a study by Katz and Rotstein of 42,292 patients with osteoporosis, 754 were found to have periapical lesions (1.78%). The percentage of patients with periapical changes averaged 0.52% and was more than three times lower. The data also showed statistically significant differences between patients with and without osteoporosis. When osteoporosis is also associated with bisphosphonate intake, the percentage of patients with periapical lesions is lower. The type of medication is also important. Treatment with alendronate and risedronate resulted in statistically significant differences compared to other patients with osteoporosis. 43

Cadoni et al found that osteoporosis could not be associated with the appearance of periapical lesions, although they were more common in these patients. Their increased frequency during root canal treatment highlights the possible connection between the dynamics of the healing process and osteoporosis. The beneficial influence of bisphosphonates is emphasized and further research in this direction is recommended. 44

The intake of bisphosphonates has such a strong influence on the bone structure that it is part of the criteria for excluding patients in the study of the postextraction alveolus. 45 This is of particular importance in surgical manipulations and placement of implants. People with osteoporosis belong to the group of medically compromised patients. With them, it is necessary to provide appropriate consultation and premedication in order for the treatment to be successful. 46 In cases of primary osteoporosis, implant treatment is not contraindicated, despite the changes in the bone structure. 47

Yarbasi and Bozdemir recommended that before starting treatment with bisphosphonates in patients with osteoporosis, the oral health should be assessed and the patient's teeth should be treated. It is important that they are trained to practice proper oral hygiene and perform preventive examinations. In addition to the standard tests, they should also be monitored for signs of osteonecrosis. This type of patient should be treated jointly by dentists and general practitioners. 48

The dental treatment of patients taking bisphosphonates has some peculiarities. However, the knowledge of dentists is dentists is unsatisfactory. 49

### Bisphosphonates—Alendronate

Xiong et al investigated the effect of alendronate on periapical lesions and osteoporosis due to reduced estrogen levels. This study was conducted on experimental mice, and the medication was placed subcutaneously every day. This resulted in a decrease in bone loss and a decrease in the number of osteoclasts. 50 Alendronate affects osteocyte apoptosis and IL-6-related inflammatory response mechanisms. 51

Lilakhunakon et al find that alendronate reduced cell adhesion and viability of preosteoblasts on Ti surfaces. Alendronate treatment seemed to exert higher inhibitory effects on nuclear shape and size as well as cell viability in lower cell passage. This led to the reduction in cell to implant surface interaction after encountering bisphosphonate treatment. 52

### Bisphosphonates—Zoledronate

Wayama et al investigated the effects of another bisphosphonate, ZOA, in mice. He uses microcomputed tomography in his research. Several groups are studied: a control group receiving a placebo, ovariectomy group receiving placebo, a control group receiving ZOA, and an ovariectomized group receiving ZOA. The results show that taking ZOA can reduce bone destruction in patients with low estrogen levels. 53 The results of Ikeda et al confirm that zoledronic acid suppresses periapical bone resorption with maintained osteoclast numbers. 19 Maia et al also proved the potential of zoledronic acid in patients with osteoporosis, noting its anti-inflammatory and antiresorptive effect on bone. 54

Marques-Ferreira et al found that in patients without osteoporosis and the presence of zoledronate therapy, periapical lesions had lower bone resorption compared to the control group. 55

### Calcitonin

In these patients, the hormone calcitonin can be administered. It inhibits bone resorption, but has a weaker effect than bisphosphonates and is therefore rarely used in the treatment of osteoporosis. 56

### Raloxifene

Another medicinal product that is used for osteoporosis is raloxifene. Gomes-Filho et al investigated the mechanisms of osteoclast formation and angiogenesis in periapical lesions of mice with reduced estrogen levels. In their study, they used two groups of animals, one receiving raloxifene and the other a placebo. The results showed higher levels of alkaline phosphatase, calcium, and phosphates in the group of mice that received raloxifene compared to the control group. This gives the authors reason to suggest this medication as part of the treatment for patients with reduced estrogen levels. 57

### Follicle-Stimulating Hormone and Leuprorelin

Liu et al investigated the effect of follicle-stimulating hormone and its inhibitor leuprorelin on mice with periapical lesions. The results showed that leuprorelin significantly reduced bone loss and the number of osteoclasts compared to the control group. 58 There are similar results in the study by Qian et al: Increased expression of follicle-stimulating hormone and secretion of proinflammatory cytokines, such as IL-1β, IL-6, and TNF-α, affect periodontal ligament cells; increased production of pro-inflammatory cytokines induced by *Porphyromonas gingivalis* lipopolysaccharide. High levels of follicle-stimulating hormone may regulate the immune status of postmenopausal periodontal tissues. 59

### Strontium

Strontium compounds (strontium ranelate) are also used to treat osteoporosis. The mechanism of action is not fully understood, but it is assumed that calcium receptors are activated, cyclooxygenase-2 and prostaglandin E2 are induced. Adverse reactions are associated with diarrhea and headache. 60

Although there are no data on the effect of strontium preparations against osteoporosis on periapical lesions, Oztekin et al found that strontium nanoparticles could significantly reduce bone resorption and promote new bone formation with a higher frequency of osteoblasts. The authors note the potential of strontium compounds in the treatment of periapical lesions. 61

### Denosumab

Another medication to treat osteoporosis is the monoclonal antibody denosumab. It reduces the risk of fractures. It acts as an inhibitor of RANKL expression. It is effective against bone loss by increasing bone density. However, there is evidence that it leads to an increased risk of infections and endocarditis and skin disease. 62 With tooth extractions, there is a serious risk of denosumab-associated necrosis of the jaw when the drug is used to treat cancer. 63

### Parathyroid Hormone

Parathyroid hormone may also be administered during treatment. Because of its high cost, it is used only in severe cases. 6 This hormone protects the periapical tissues and the periapical area when it is used. 64

### Combined Therapy

In some cases, patients with osteoporosis take both corticosteroids and bisphosphonates. They can also be successfully treated with endodontics. In Lajla's clinical case, the patient was taking corticosteroids (Medrol 4 mg) and bisphosphonates (ibandronic acid). Besides osteoporosis, he has other common diseases: Sjogren's syndrome and hypothyroidism. Additionally, he suffers from temporomandibular joint movement disorders. The diagnosis was periapical abscess with fistula. In the treatment, temporary medication with calcium hydroxide and chlorhexidine (gel) is applied three times a month, after which the root canals are definitively obturated. 65

This clinical case, as well as the mentioned studies, shows the relationship between apical periodontitis and osteoporosis. This is one of the examples where common chronic diseases influence the course of apical periodontitis. 66

## Conclusion

A review of the specialized literature shows that current research confirms the relationship between chronic diseases and periapical lesions. It is well manifested and documented in patients with osteoporosis. When treating patients with periapical lesions, the dentist must take a proper medical history and general medical history. It is good to keep in mind the reduced bone density when reading the patient's X-rays.

On the other hand, an X-ray examination in connection with dental treatment may be the reason for the detection of osteoporosis in patients. In cases of osteoporosis or taking bisphosphonates and other medications, consideration should be given to whether consultation with a specialist is necessary, what treatment approach would be most appropriate, and what the prognosis will be.
